# HARNESSING PROTEIN INTAKE AND MICROBIOME INTERACTIONS TO IMPROVE HEALTHSPAN

**DOI:** 10.1093/geroni/igaf122.1799

**Published:** 2025-12-31

**Authors:** Cristal Hill, Prerana Vaddi

## Abstract

Substantial evidence demonstrate that gut dysbiosis affects metabolic health, particularly while advancing in age. Notably, females tend to experience greater age-related comorbidities such as diabetes and neurodegeneration, many of which are driven by metabolic dysfunction and cellular senescence. Previous work from our lab has demonstrated that dietary protein restriction (DPR) induces fibroblast growth factor 21 (FGF21) to improve metabolic health and extend lifespan in male mice. However, the effects of DPR on gut health in aged female mice remains unclear. In this study, we investigate how DPR influences metabolic health and the gut microbiome in aged female mice. Female mice subjected to DPR at 16 months until 22 months of age exhibited notable improvements in metabolic health. Relative abundances of Clostridium were reduced, while Faecalibaculum rodentium and Kineothrix were increased. Gene set enrichment analysis (GSEA) revealed that bacterial groups associated with membrane integrity and metal ion binding were most enriched during DPR.

